# Incidence of Gastric Metaplasia and *Helicobacter pylori* Infection in Duodenal Bulb – With Specific Reference to Patients With Duodenal Ulcers

**DOI:** 10.1155/DTE.6.17

**Published:** 1999

**Authors:** Minoru Kawaguchi, Toshihiko Saito

**Affiliations:** Fourth Department of Internal Medicine Tokyo Medical University Japan

## Abstract

We determined the incidence of gastric metaplasia in the duodenal bulb of duodenal ulcer patients and the *Helicobacter pylori* (*H. pylori*) infection rate at sites with gastric metaplasia. Biopsy of the duodenal bulb showed the presence of gastric metaplasia in 61 of 86 patients (71%) overall and in 18 of 47 patients (38.3%) who had gastrectomy at an early gastric cancer. The histological diagnosis of *H. pylori* infection showed good agreement (83.3%) with the result of the rapid urease test, indicating that *H. pylori* occurs in regions with gastric metaplasia. This finding suggests that *H. pylori* infects gastric metaplasia in the duodenal bulb, causing mucosal injury, which is then transformed into duodenal ulcers. The exact mechanism by which gastric metaplasia is caused is unknown, but it is believed to occur in the transitional zone in the duodenal mucosa.


*Diagnostic and Therapeutic Endoscopy*, Vol. 6, pp. 17–23
Reprints available directly from the publisher
Photocopying permitted by license only

© 1999 OPA (Overseas Publishers Association) N.V.
Published by license under
the Harwood Academic Publishers imprint,
part of The Gordon and Breach Publishing Group.
Printed in Malaysia.

### Incidence of Gastric Metaplasia and *Helicobacter pylori* Infection in Duodenal Bulb – With Specific Reference to Patients with Duodenal Ulcers

MINORU KAWAGUCHI* and TOSHIHIKO SAITO

*Fourth Department of Internal Medicine, Tokyo Medical University, Japan*

*(Received 21 January 1999; Revised 2 April 1999; In final form 24 May 1999)*

**We determined the incidence of gastric metaplasia in the duodenal bulb of duodenal ulcer patients and the *Helicobacter pylori* (*H. pylori*) infection rate at sites with gastric metaplasia. Biopsy of the duodenal bulb showed the presence of gastric metaplasia in 61 of 86 patients (71%) overall and in 18 of 47 patients (38.3%) who had gastrectomy at an early gastric cancer. The histological diagnosis of *H. pylori* infection showed good agreement (83.3%) with the result of the rapid urease test, indicating that *H. pylori* occurs in regions with gastric metaplasia. This finding suggests that *H. pylori* infects gastric metaplasia in the duodenal bulb, causing mucosal injury, which is then transformed into duodenal ulcers. The exact mechanism by which gastric metaplasia is caused is unknown, but it is believed to occur in the transitional zone in the duodenal mucosa.**

*Keywords:* Duodenal ulcer, Gastric metaplasia, *Helicobacter pylori*

### INTRODUCTION

The fact that the incidence of *Helicobacter pylori* (*H. pylori*) infection is high among duodenal ulcer patients and that the duodenal ulcer recurrence rate decreases following its eradication indicate that *H. pylori* is related to the pathogenesis of duodenal ulcers [1], but whether it plays a role in the development of duodenal ulcers is unknown. According to Wyatt *et al.*, *H. pylori* infection is closely related to the development of duodenal ulcers [2]. She reported that *H. pylori* adheres to gastric metaplastic cells in patients with gastric metaplasia in the duodenal bulb, causing local *H. pylori* infection, which results in injury and ulcers in the duodenal mucosa. However, only a few studies have been conducted regarding the incidence of gastric metaplasia in the duodenal bulb and the presence of *H. pylori* in regions with gastric metaplasia [3,4].

The present study was undertaken to determine the incidence of gastric metaplasia and the presence of *H. pylori* in the duodenal bulb in duodenal ulcer patients, as well as to determine the origin of gastric metaplastic cells.

* Corresponding author.

### SUBJECTS

Included in the present study were 86 patients in whom the diagnosis of duodenal ulcers or their scars was endoscopically established at the Endoscopy Center of the Tokyo Medical University and in whom a biopsy was taken from the duodenal bulb under direct vision. Of the 86 patients (mean age, 45.5; male : female ratio, 65 : 21), 37 had duodenal ulcers and 49 duodenal ulcer scars. The incidence of gastric metaplasia in the duodenal bulb was also determined in 47 patients in whom early gastric cancer was excised (control). The type of gastric cancer was IIc in 36 patients, IIb in 5 patients, IIa in 5 patients and I in 1 patient (mean age 58.9; male : female ratio, 43 : 4).

### METHODS

Biopsies taken from the duodenal bulb under direct vision in patients with duodenal ulcers or their

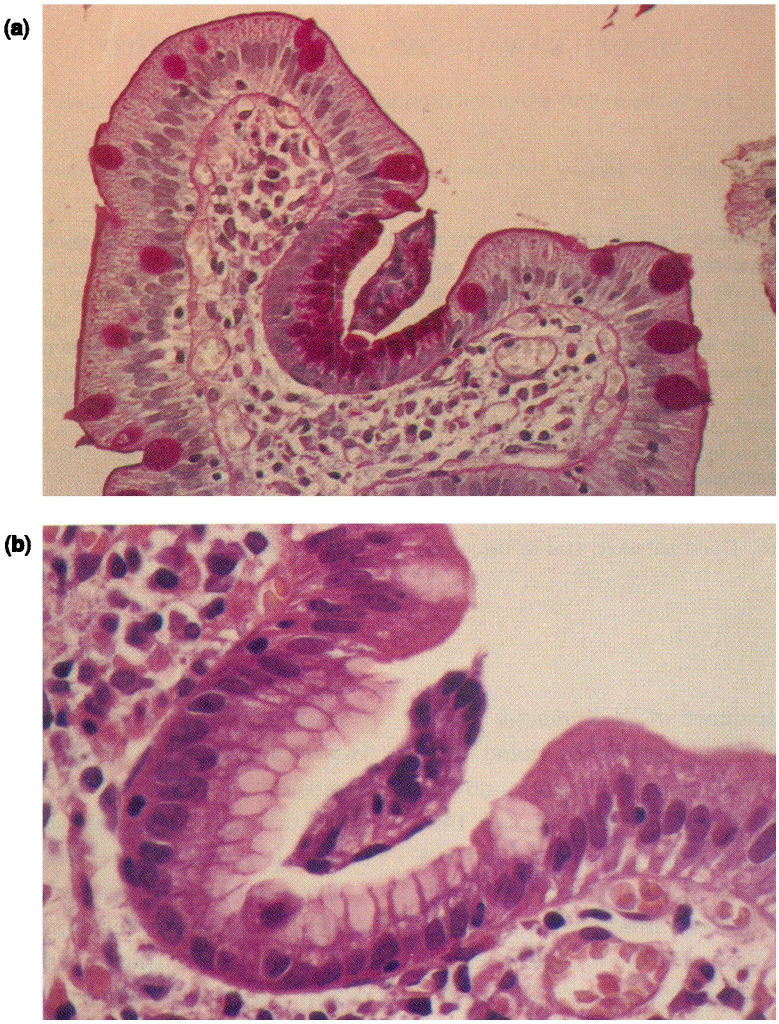

FIGURE 1 Biopsy taken from the duodenal bulb (a) AB–PAS staining ×200; (b) HE staining ×400. Gastric metaplastic cells in the center are PAS-positive and have no brush border.

scars and those with early gastric cancer were stained with hematoxylin and eosin (HE) and Alcian Blue–periodic acid Schiff reaction (AB–PAS). Biopsy specimens were taken every one specimen from about 5 mm distance away from the ulcers or scars. The diagnosis of gastric metaplasia was made if PAS-positive cells were observed in the absence of brush borders (Fig. 1). The presence of *H. pylori* was confirmed by Giemsa staining, the urease test (CLO test) and culture.

### RESULTS

1. Examinations of biopsies taken under direct vision from 86 patients with duodenal ulcers or their scars confirmed the presence of gastric metaplasia in 61 patients (male : female ratio, 47 : 14) (71%) (Table I).
2. Gastric metaplasia was observed in the duodenal bulb mucosa in 18 (male : female ratio, 16 : 2) of the 47 patients (38.3%) with excised early gastric cancer (Table I).
3. In 12 patients (9 men and 3 women; mean age, 45.8), gastric metaplasia was confirmed by biopsy of the duodenal bulb under direct vision and the rapid urease test (CLO test) was conducted with biopsies taken from the same site at the same time. Biopsy samples were subjected to Giemsa staining, and the presence or absence of *H. pylori* was histologically studied. The presence of *H. pylori* was confirmed in 7 of the 12 patients (58.3%). The presence or absence of *H. pylori* and the results of the urease test coincided in 83.3% of patients (Table II).

TABLE I Incidence of gastric metaplasia in the duodenal bulb

| | | |
|---|---|---|
| Duodenal ulcer/scar (biopsy) | 61/86 | 71%* |
| Early gastric cancer (resected) | 18/47 | 38.3%* |

*$p = 0.001$.

TABLE II Coincidence of the results of histological examinations and the urease test regarding the presence of *H. pylori* in the duodenal bulb

| Urease test | Histological examination | | Total |
|---|---|---|---|
| | + | – | |
| + | 5 | 0 | 5 |
| – | 2 | 5 | 7 |
| Total | 7 | 5 | 12 |

### DISCUSSION

Various studies conducted since the discovery of *H. pylori* by Warren and Marshall in 1983 [5] showed that *H. pylori* infection is closely related to various gastric and duodenal diseases. The fact that the incidence of *H. pylori* infection is high among duodenal ulcer patients and that the duodenal ulcer recurrence rate decreases following its eradication indicate that *H. pylori* is closely related to duodenal ulcer [6,7], but whether it is an etiological factor for duodenal ulcers is unknown. Wyatt *et al.* proposed that gastric metaplasia is caused during the repairing process of erosion caused to the duodenal bulb, that gastric metaplasia cells are infected by *H. pylori*, that inflammation occurs in the infected area, and that this results in mucosal injury and ultimately ulcers.

In the present study, we investigated the incidence of gastric metaplasia in the duodenal bulb and determined whether gastric metaplasia is infected by *H. pylori* using biopsies taken from the duodenal bulb mucosa under direct vision in patients with duodenal ulcers or their scars who gave informed consent. An atypical epithelium observed in the duodenal bulb includes the ectopic gastric mucosa which consists of almost normal fundus gland mucosa (Fig. 2) and gastric metaplasia observed in the regeneration process following erosions (Fig. 3). Under an endoscope, ectopic gastric mucosa presents whitish small elevated lesions (Fig. 4) and is seldom observed in the duodenal bulb of duodenal ulcer patients, indicating that it plays almost no role in the etiology of duodenal ulcers. Gastric

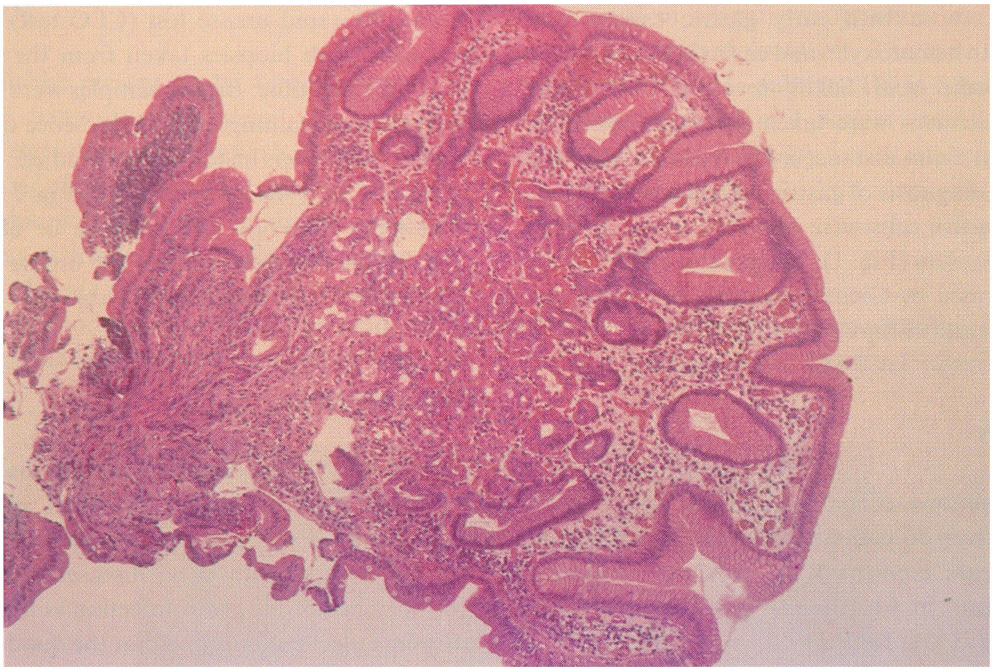

FIGURE 2 Biopsy taken from the duodenal bulb (HE staining ×40). The ectopic gastric mucosa consists of almost normal fundic gland mucosa.

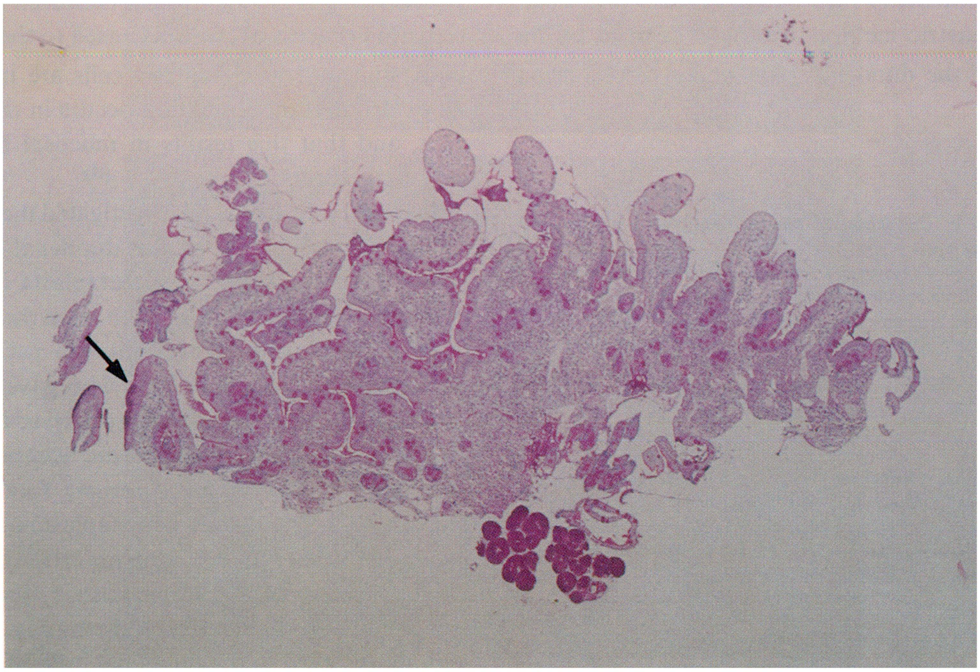

FIGURE 3 Biopsy taken from the duodenal bulb in a patient with a duodenal ulcer scar (AB–PAS staining ×40). Gastric metaplasia is observed in the small arrowed area.

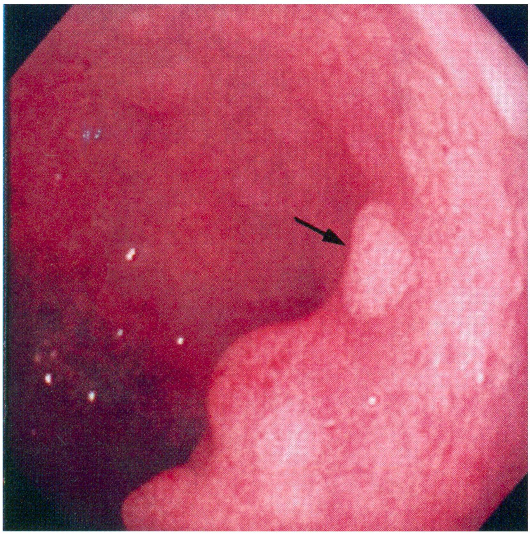

FIGURE 4 Endoscopic picture of the duodenal bulb. The arrow indicates ectopic gastric mucosa.

metaplasia is believed to be produced in the regeneration process from erosions caused to the duodenal bulb, but there are a few reports on the origin of gastric metaplastic cells. In the present study, we observed transitional regions (Fig. 5) between these cells and duodenal glands in many specimens and thought that differentiation into gastric metaplastic cells from these regions, suggesting that the gastric metaplastic cell occurs during regeneration from erosions. However, whether cells in these regions differentiate into gastric metaplastic cells on the surface and duodenal glands in a deeper area or whether the duodenal glands differentiate into gastric metaplastic cells is unknown.

Gastric metaplasia was observed in the duodenal bulb of a high percentage (71%) of patients with duodenal ulcers or their scars. The duodenal bulb of patients with excised early gastric cancer with no history of duodenal ulcer was histologically evaluated as control. (The absence of duodenal ulcers or their scars was confirmed in examined specimens.) Gastric metaplasia was observed in 18

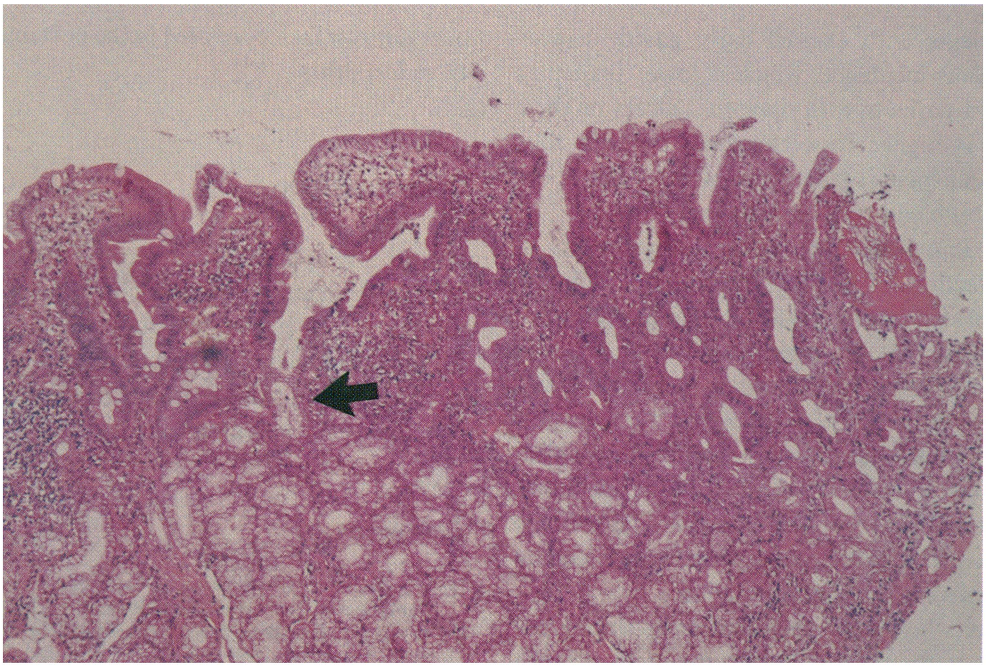

FIGURE 5 Biopsy taken from the margin of the duodenal ulcer (HE staining ×40). Transition from duodenal glands to gastric metaplastic cell is observed in the arrowed area.

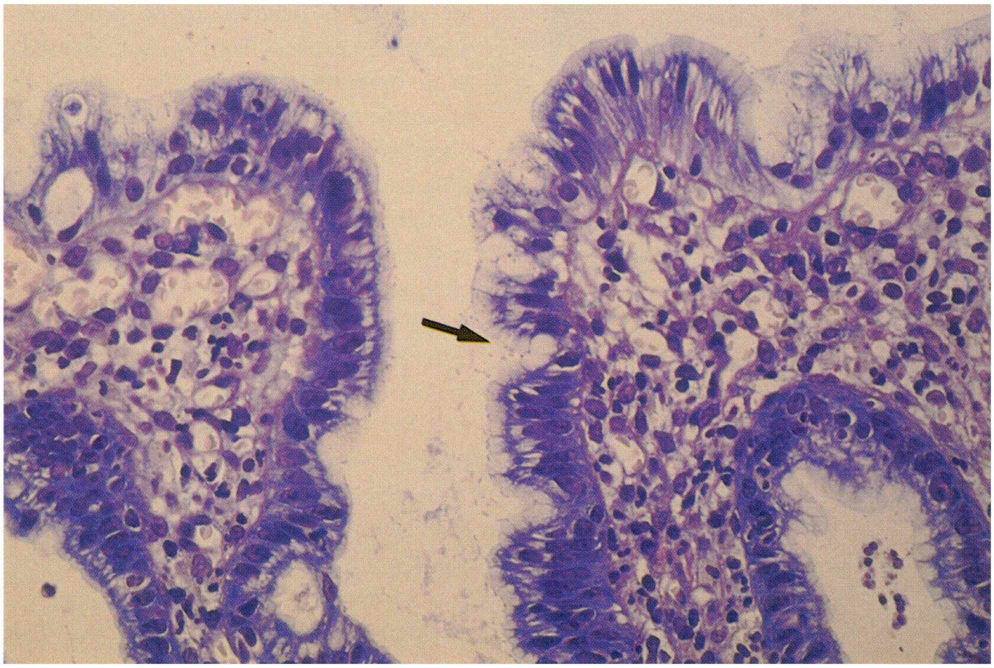

FIGURE 6 *H. pylori* is present in the arrowed gastric metaplastic region (Giemsa staining ×400).

of 47 patients with excised early gastric cancer (38.3%). This incidence, which is lower than that observed in patients with duodenal ulcers or their scars but is higher than expected, suggests that erosions and their regeneration often occur in the duodenal bulb without ulcers as well.

To our knowledge, there are a few reports in which infection of gastric metaplasia cells by *H. pylori* was confirmed. In the present study, we demonstrated that the presence of *H. pylori* was found to be present in regions with gastric metaplasia (Fig. 6) in 58.3% of patients. The presence or absence of *H. pylori* determined by histological examinations coincided with results of the rapid urease test in 83.3% of cases. These findings indicate that regions with gastric metaplasia are infected by *H. pylori* which causes inflammation.

However, how *H. pylori*-infected duodenitis progresses to duodenal ulcers is unknown as is the mechanism by which *H. pylori*-infected gastritis progresses to gastric ulcers. Further studies are necessary on factors related to the host and different *H. pylori* strains.

### CONCLUSION

Gastric metaplasia often occurs in the duodenal bulb, and regions with gastric metaplasia are often infected by *H. pylori*, which causes inflammation. Gastric metaplasia is thought to occur in the process of regeneration from erosions and is differentiated from transitional regions.

### References

[1] Goodwin, C.S. Duodenal ulcer, *Campylobacter pylori* and the "leaking roof" concept. *Lancet* 1988; **2**: 1467–1469.
[2] Wyatt, J.I., Rathbone, B.J., Dixon, M.F. *et al. Campylobacter pylorides* and acid-induced gastric metaplasia in the pathogenesis of duodenitis. *J. Clin. Pathol.* 1987; **40**: 841–848.
[3] Urakami, Y., Kimura, M. and Seki, H. Gastric metaplasia and *Helicobacter pylori*. *Am. J. Gastroenterol.* 1997; **92**: 795–799.

[4] Satoh, K., Kimura, M., Yoshida, Y. *et al.* Relationship and inflammation of the duodenal mucosa. *Am. J. Gastroenterol.* 1993; **88**: 360–363.

[5] Warren, J.R. and Marshall, B.J. Unidentified curved bacilli on gastric epithelium in active chronic gastritis. *Lancet* 1983; **1**: 1273–1275.

[6] NIH Consensus. Development Panel on *Helicobacter pylori* in Peptic Ulcer Disease. *Helicobacter pylori* in peptic ulcer disease. *JAMA* 1994; **272**: 65–69.

[7] Asaka, M., Ohtaki, T., Katou, M. *et al.* Causal role of *Helicobacter pylori* in peptic ulcer relapse. *J. Gastroenterol.* 1994; **29**(Suppl. 7): 134–138.

